# Comprehensive Analysis of the Aberrant Right Subclavian Artery: A Perspective from a Single Institute

**DOI:** 10.3390/diagnostics15060772

**Published:** 2025-03-19

**Authors:** Rou Jiun Lin, Kim-Seng Law, Pei-Jhen Wu

**Affiliations:** 1 Department of Obstetrics and Gynecology, Tung’s Taichung Metroharbor Hospital, 699 Taiwan Boulevard, Taichung 435, Taiwan; d4320@ms3.sltung.com.tw; 2 Department of Post-Baccalaureate Medicine, National Chung Hsing University, 145 Xingda Road, Taichung 402, Taiwan; 3 Department of Medical Research, Tung’s Taichung Metroharbor Hospital, 699 Taiwan Boulevard, Taichung 435, Taiwan; judy456789judy@gmail.com

**Keywords:** aberrant right subclavian artery, genetic abnormalities, karyotyping, ultrasound, prenatal diagnosis

## Abstract

**Background/Objectives**: This study aimed to provide a descriptive review of fetal aberrant right subclavian artery (ARSA), with a discussion of the genomic and structural anatomy and perinatal prognosis in our hospital’s obstetric department. **Methods**: In total, 3266 fetal level II sonographies were performed between January 2020 and June 2023. The 21 cases diagnosed with ARSA were included in this study. Obstetric ultrasound screening, noninvasive prenatal screening, and fetal karyotyping were performed. Fetal echocardiograms, postnatal information, and follow-up data were recorded. **Results**: In our dataset of 3266 cases, the overall incidence rate of ARSA was 0.6%. Of the 21 fetuses with ARSA, no abnormalities were detected in either prenatal or genetic tests, and no chromosomal anomalies were identified. **Conclusions**: Our study provides informative insights into ARSA, emphasizing the need for a comprehensive evaluation of its structural and genetic aspects. The findings of this study prompt further exploration, especially regarding the increasing incidence of ARSA and the potential role of advanced genetic analyses in enhancing diagnostic precision and fetal prognostic evaluation.

## 1. Introduction

An aberrant right subclavian artery (ARSA) emerges as a consequence of abnormal development in the primitive aorta and aortic arches, presenting as a distinctive anatomical variation observed in approximately 1–2% [1] of the general population. This anomaly manifests when the distal segment of the right dorsal aorta and the seventh intersegmental artery collaboratively contribute to the formation of the right subclavian artery [2]. This intricate process involves the fourth right aortic arch and proximal section of the dorsal aorta, resulting in a diverse range of presentations.

The course and origin of an ARSA are characterized by unique anatomical features. Typically, the right subclavian artery is the first vessel to branch from the brachiocephalic trunk, which is the major branch of the aortic arch. An ARSA can directly originate from the aortic arch, coursing behind the trachea toward the right arm. Additionally, it may traverse the posterior aspect of the esophagus and trachea or even directly arise from the descending aorta. This anomalous vascular pattern results from the complex interplay between the distal right dorsal aorta and the seventh intersegmental artery during embryonic development at the third gestational age.

The typical vascular configuration includes three branches: the right common carotid, the left common carotid, and the left subclavian arteries. However, when an ARSA coexists with the left aortic arch, four branches originate from the left aortic arch. This specific configuration, featuring a left aortic arch with an ARSA, is recognized as one of the most prevalent anomalies in the arch division process. Another distinct variation, termed the aberrant retroesophageal right subclavian artery, follows a course from the left side of the spine, passing behind the esophagus and trachea before reaching the right upper arm [3].

Understanding the intricate anatomical details of an ARSA, including its diverse presentations and associations with left aortic arch anomalies, is crucial for understanding the complexity of this vascular anomaly. Such insights will contribute to a more thorough understanding of embryonic vascular development and aid in the clinical interpretation of aberrations in the aortic arch system.

ARSA is often associated with congenital aortic arch anomalies. A study by Stavridis et al. (2022) [4] consistently linked ARSA to chromosomal abnormalities, particularly trisomy 21. The reported prevalence of ARSA in patients with trisomy 21 widely varies, ranging from 2.8% to 100%, as indicated by pathological and postnatal studies [5,6,7,8,9,10,11,12], leaving the true incidence uncertain.

ARSA can be categorized as isolated and non-isolated. The non-isolated variant, especially when accompanied by high-risk first-trimester screening, structural defects, and soft markers, demonstrates a higher incidence of chromosomal abnormalities. Cardiac anomalies, such as ventricular septal defects, pulmonary stenosis, and the left aortic arch, are the structural abnormalities frequently associated with ARSA, reaching an incidence rate of 16%, as reported by Borenstein et al. [13,14,15].

Isolated ARSA, defined as ARSA occurring in a fetus with no other structural or soft marker findings, is not associated with an increased risk of aneuploidy or identifiable markers. Consequently, the association between isolated ARSA and Down, Turner, and DiGeorge syndromes remains unclear [1,11,12,16,17,18,19]. Given its association with certain chromosomal defects, ARSA may serve as a valuable marker, particularly when used in conjunction with other ultrasound markers, as highlighted by Borenstein et al. (2010) [15]. Advanced maternal age (AMA) is also a concern, and a study by Chen et al. emphasized the high prevalence of chromosomal abnormalities in fetuses with ARSA born to women with AMA [14].

This retrospective study aimed to assess sonographic findings and explore the potential correlation between an ARSA and chromosomal abnormalities.

## 2. Materials and Methods

### 2.1. Study Population and Device

Our study comprised 3266 patients undergoing routine level II sonography check-ups at 21–23 weeks of gestational age, focusing specifically on 21 singleton pregnancies with right subclavian artery anomalies. The assessments included obstetric ultrasound screening, noninvasive prenatal screening, and amniocentesis. Participants aged 24–39 years were included in this study. Our dataset provides comprehensive information on routine fetal antenatal ultrasound screening, noninvasive prenatal testing, and fetal karyotype analysis. Neonatal follow-up care was consistently provided in all patients.

**Figure 1:**
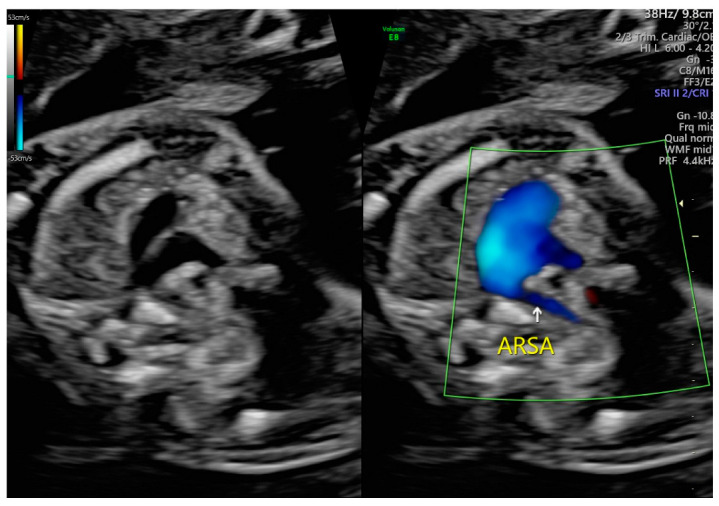
Color Doppler ultrasound images showing a fetal aberrant right subclavian artery at 22 weeks of gestation.

**Figure 2:**
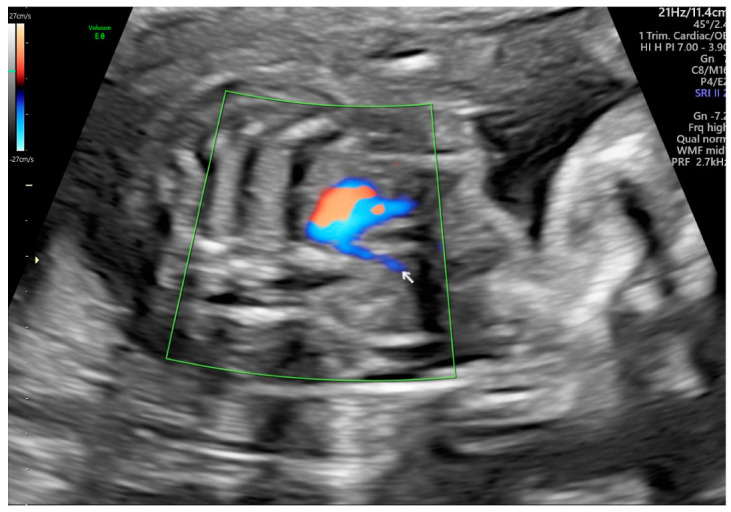
Color Doppler ultrasound images of a fetal aberrant right subclavian artery at 21 weeks of gestation. The arrow point to the site of ARSA.

ARSA cases were identified during a routine prenatal transabdominal ultrasonography of unselected patients at our hospital between July 2020 and June 2023. Grayscale and color Doppler ultrasonography in the transverse three-vessel view was used to assess the right subclavian arteries. The presence of ARSA in the transverse plane was meticulously confirmed by two operators with specialized experience using the GE Voluson E8 and S10 ultrasound machines (Waukesha, WI, USA). The examination was performed during the second trimester at a gestational age ranging between 21 and 23 weeks and adhered to the International Society of Ultrasound in Obstetrics and Gynecology guidelines for structures and detection techniques. The incidence of ARSA in the second trimester ranges from 0.4% to 1.5% in chromosomally normal fetuses [20]. The examination used a B-mode segmental view approach complemented by color Doppler ultrasonography with specific color Doppler velocity settings to enhance clarity. The ultrasonic findings are presented in Figure 1 and Figure 2 for reference.

### 2.2. Karyotyping Analysis

All participants were provided with the option to select either noninvasive prenatal testing (v1.0, v2.0, v3.0) or invasive karyotype analysis. The invasive testing procedures included amniotic fluid karyotyping and array-based comparative genomic hybridization analysis.

### 2.3. Postnatal Assessment

Additional case details, postnatal echocardiogram findings, and follow-up data in the pediatric outpatient department were also recorded.

## 3. Results

Between January 2020 and June 2023, we assessed 3266 pregnant women in their second trimester who opted for level II sonography examination. ARSAs were detected in 21 (0.6%) patients. In our analysis of these 21 patients, the prenatal detection of ARSA occurred in singleton pregnancies, with a mean maternal age of 31.6 years, with infants born from 29 weeks and 1 day to 39 weeks and 4 days (mean gestational age, 38 weeks and 2 days). Among the participants, 33% were mothers aged ≥35 years. The sex distribution of the fetuses was nearly equal, with 52% females and 47% males. The outcomes revealed that 95% of the fetuses were alive, whereas 4% were stillbirths (Table 1).

Among the 21 fetuses with ARSA, the observations were as follows (Table 2).

- Amniocentesis and single-nucleotide polymorphism (SNP) microarray testing: Nine of the 21 patients underwent amniocentesis, and four underwent further SNP microarray testing. The procedure was carried out in three patients after the finding of ARSA.
- Noninvasive prenatal testing (NIPT): Ten of the 21 patients opted for NIPT, with two selecting NIPT1 (v1.0), five NIPT2 (v2.0), and three NIPT3 (v3.0).
- Maternal serum screening tests: Two patients underwent second-trimester Down’s syndrome screening using maternal serum tests.

Moreover, all patients with ARSA were isolated. No extracardiac anomalies were present, no chromosomal abnormalities were detected in either prenatal or genetic tests, and no other cardiac malformations were identified during clinical follow-up observations.

All live births (20/21) demonstrated a favorable prognosis, with 20 children born alive. One stillbirth occurred at 37 weeks of gestation. Not all babies received post-natal echocardiography, with the screening conducted in 12 patients revealing the presence of either a patent foramen ovale or an atrial septal defect (secundum type) with a left-to-right shunt (Table 3).

Further analysis did not include cases of stillbirth, and the remaining eight were lost to follow-up, including assessments in the postnatal outpatient department.

The subsequent outpatient department follow-up of the 12 patients involved sonographic examinations, which showed normal left ventricular systolic function and a normal heart size. The pediatricians recommended additional evaluations within a 3–12-month timeframe. Among these 12 cases, 75% (9 cases) of the infants displayed no apparent respiratory distress or cardiac issues, aside from the grade I–II short systolic murmurs detected during physical examinations. These infants exhibited normal growth during postnatal follow-up. However, follow-ups for two fetuses (16%) remained unsuccessful, warranting ongoing monitoring and assessment.

## 4. Discussion

In our dataset of 3266 cases collected between 2020 and 2023, 21 patients were diagnosed with an ARSA, resulting in an ARSA rate of 0.6% (21/3266). A further analysis based on year revealed an increasing trend, with five cases in 2020, three in 2021, seven in 2022, and seven in the current year. This increase is attributed to the growing prevalence of mothers undergoing level II ultrasound screening annually, a noninvasive diagnostic test facilitated by trained and experienced operators. Although these findings provide valuable insights into the clinical landscape of ARSA within our hospital’s obstetric practice, larger studies are required to analyze and clarify the relationship between these increasing incidences.

Song et al. observed a higher incidence of ARSA in females than in males [3]. In contrast, our study indicated an equal sex distribution in ARSA.

An ARSA is generally considered an asymptomatic and benign condition. However, cases of esophageal compression leading to dysphagia, documented by Corbacioglu et al. (2017) and Polguj (2014), underscore the potential complications [21,22]. Furthermore, the first reported case of ARSA in fetuses with Down syndrome, documented in 2005 by Chaoui et al. [23], highlights the importance of the prenatal diagnosis of this benign anomaly in aortic arch branching. Therefore, ARSA has gained recognition as a common soft ultrasound marker in the medical literature.

### 4.1. Prenatal Diagnostic Techniques and Standardization

The reliability of ARSA diagnosis is heavily dependent on the imaging modalities and the expertise of the operator. Currently, the standard approach involves second-trimester two-dimensional (2D) ultrasonography with color Doppler, which, despite its widespread use, may be limited by the expertise of the operator and the suboptimal spatial resolution. In recent years, three-dimensional (3D) ultrasonography and fetal magnetic resonance imaging (MRI) have emerged as promising adjuncts that could enhance the detection and characterization of vascular anomalies. For instance, 3D imaging enables a more detailed visualization of the aortic arch and branching patterns, potentially reducing the number of false negatives. However, the integration of these advanced imaging techniques into routine clinical practice necessitates the development of standardized protocols and training modules. Establishing consensus guidelines regarding image acquisition, interpretation, and reporting would not only improve the diagnostic accuracy but also facilitate inter-center comparisons in multicenter studies. In addition, future research should address the cost-effectiveness and feasibility of incorporating these advanced imaging modalities into standard prenatal care, especially in resource-limited settings.

Despite the increasing recognition of ARSA, studies exploring its genetic etiology are limited. The existing literature reveals conflicting data regarding the association between isolated ARSA and chromosomal abnormalities, particularly trisomy 21, leading to a lack of consensus regarding the recommendation of karyotyping for all low-risk and isolated ARSA pregnancies [24]. Consequently, the need for invasive tests in these cases remains controversial. Studies have also suggested that isolated ARSA has a low likelihood of being linked to pathogenic copy number variations (CNVs). Nevertheless, discussions led by Cai et al. [2] introduced a nuanced perspective by suggesting that when ARSA coincides with supplementary ultrasound abnormalities, the risk of pathogenic CNV may increase. Thus, they recommended the inclusion of prenatal genetic counseling and SNP array testing to evaluate the fetal prognosis.

In our cases, ARSA was identified as the sole abnormal ultrasound finding, with no concurrent soft marker findings or chromosomal abnormalities. This clinical profile suggests that these cases can be categorized as isolated ARSA. Importantly, our findings align with the existing literature, indicating that isolated ARSA does not exhibit a statistically significant association with an increased risk of aneuploidy. Nevertheless, it is crucial to note the occurrence of one case that resulted in a stillbirth diagnosed as intrauterine fetal demise. The etiology of stillbirth remains undetermined, underscoring the complexity and multifactorial nature of the adverse outcomes associated with ARSA.

Nevertheless, our study has certain limitations that merit careful consideration. First, the limited sample size may restrict the broader applicability of our findings. The ultrasound diagnosis of ARSA relied solely on the three-vessel and trachea views, which focus on key structures, such as the aorta, pulmonary artery, superior vena cava, and trachea. Although these views are foundational, the absence of a coronal view may have hindered optimal spatial visualization and the comprehensive assessment of the vascular anatomy.

The occurrence of one intrauterine fetal death, resulting in a mortality rate of 4% (1/21), suggests that there may be associations with chromosomal abnormalities; however, it is unclear whether these incidents are purely coincidental. Moreover, not all the participants had invasive karyotype abnormalities, which introduced a potential source of bias. The influence of genetic abnormalities remains unclear, emphasizing the need for further studies and comprehensive studies.

### 4.2. Association with Other Congenital Cardiovascular Anomalies

Although ARSA is most commonly identified as an isolated anatomical variant, emerging evidence suggests that it may coexist with other congenital cardiovascular abnormalities. Several studies have noted associations between ARSA and structural heart defects such as ventricular septal defects (VSDs), pulmonary stenosis, and atrial septal defects (ASDs), albeit with varying incidence rates. It is imperative to consider that the presence of ARSA might serve as an additional sonographic marker in cases where other cardiovascular malformations are suspected. In particular, non-isolated ARSA cases—especially those identified in the context of high-risk first-trimester screening or multiple soft markers—may warrant more comprehensive echocardiographic evaluations. Future research should aim to systematically characterize the co-occurrence of ARSA with other congenital heart defects, using large-scale, multicenter cohorts to establish clearer epidemiological and clinical correlations. This approach would facilitate a better understanding of the spectrum of cardiovascular anomalies that may accompany ARSA and help in the development of tailored diagnostic protocols.

### 4.3. Molecular Genetics and Genomic Considerations

The genetic underpinnings of ARSA remain an area ripe for further investigation. Recent advances in molecular genetic techniques, including array comparative genomic hybridization (array CGH), single-nucleotide polymorphism (SNP) arrays, and next-generation sequencing (NGS), have the potential to elucidate subtle genomic alterations that traditional karyotyping might miss. Although our study and several others have reported a low incidence of chromosomal anomalies in isolated ARSA, the possibility of pathogenic copy number variations (CNVs) or single-gene mutations contributing to this aberrant vascular development cannot be entirely excluded. A detailed genomic analysis may uncover candidate genes or regulatory elements that are critical in the embryonic development of the aortic arch system. Furthermore, comprehensive genetic profiling could help stratify the risk, particularly in fetuses where ARSA is identified alongside other markers of congenital abnormalities. Future studies employing high-resolution genomic techniques may provide a more nuanced understanding of the molecular etiology of ARSA, ultimately leading to improved prenatal counseling and management strategies.

The increasing significance of prenatal ultrasound screening has contributed to the increased detection of ARSA using level II sonography. Recent advances in gene analysis, particularly the integration of next-generation sequencing, have markedly improved the identification of SNP and pathogenic copy number variants. This advanced approach holds promise for delivering a more precise diagnosis and enhancing the evaluation of fetal prognosis. It is noteworthy that our hospital presently exclusively provides array comparative genomic hybridization. The potential advantages of SNP analysis, with its potential to provide a higher resolution, require further exploration through comprehensive studies.

Postnatal echocardiography was performed in 12 (57%) infants, all of whom had normal postnatal development. However, three cases were lost to follow-up for unknown reasons or potentially sought care at another hospital for outpatient department follow-up. Notably, the remaining eight cases, which did not undergo fetal ultrasound screening, could not be traced.

Despite the challenges presented by the lost cases, our study demonstrates a favorable prognosis for live births. This observation supports the notion that the generally benign nature of ARSA is a congenital anomaly that may not cause symptoms in many cases, but one that can result in dyspnea, dysphagia, or problems related to heart function. The positive outcomes observed in our study contribute to our confidence in characterizing ARSA as a benign finding in most cases without other soft markers.

### 4.4. Long-Term Outcomes and Follow-Up Management

While ARSA is generally considered a benign variant, the long-term follow-up of affected individuals is essential to fully appreciate the potential clinical implications. Although our study reported favorable postnatal outcomes in most cases, there is a growing body of literature that highlights the possibility of late-onset complications such as dysphagia lusoria, respiratory difficulties, or even vascular aneurysm formation. Longitudinal studies are needed to monitor these individuals well into childhood and adulthood to determine whether isolated ARSA may predispose patients to such complications. A systematic follow-up protocol should be developed, incorporating regular cardiovascular assessments and, where indicated, advanced imaging studies to detect subclinical changes over time. Moreover, prospective registries could be instrumental in gathering comprehensive outcome data, thereby informing future guidelines on the management and surveillance of ARSA. The establishment of such protocols would also aid clinicians in counseling families regarding the expected natural history of this vascular anomaly.

In conclusion, isolated ARSA cases do not exhibit an elevated risk of aneuploidy and are devoid of soft markers or structural defects that are identifiable during anatomical surveys. However, ARSA remains a clinically valuable prenatal marker of chromosomal abnormalities if it is associated with other structural abnormalities. We recommend a comprehensive genetic workup to ensure a thorough evaluation, with no invasive amniocentesis being warranted in an isolated ARSA. Notably, this investigation was limited in its assessment of the risk of ARSA and its associations with AMA or high-risk screening. A further exploration of their relationship with pathogenic CNVs is warranted. Further studies are required to improve our understanding of this phenomenon and its clinical implications.

## Figures and Tables

**Table 1:** Demographic of maternal characteristics.

Parameter	Median (Range)/*n* (%)	
Maternal age (years)	32	(24, 39)
Height	158	(152, 167.5)
Weight	73.3	(55, 113)
BMI	27.72	(22.04, 40.28)
Gestational age	38 + 2	(29 + 1, 39 + 4)
Fetal body weight (gm)	2864	(1038, 3600)
Fetal outcome		
Alive	20	(95.23)
Stillbirth	1	(4.76)
Fetal sex		
Male	10	(47.62)
Female	11	(52.38)

**Table 2:** Screening and diagnostics methods used in affected fetus (*n* = 21).

Parameters	*n*
STS (normal)	2
Karyotype (normal)	9
Karyotype +Array (normal)	4
NIPT	10
NIPT1 (normal)	2
NIPT2 (normal)	5
NIPT3 (normal)	3

NIPT, noninvasive prenatal testing; STS: second-trimester screening.

**Table 3:** Postnatal sonographic cardiac findings (*n* = 12).

Postnatal Sonographic Cardiac Findings	*n*	%
Bilateral peripheral pulmonary stenosis	1	(8.33)
Left peripheral pulmonary stenosis	2	(16.6)
Minimal TR and mild PR with normal PA pressure	1	(8.33)
Minimal TR and minimal PR with minimal RV hypertension	1	(8.33)
Minimal TR and minimal PR with normal PA pressure	10	(83.33)
Moderate MR	1	(8.33)
Minimal MR	1	(8.33)
Muscular VSD, left-to-right shunt	1	(8.33)
No coarctation of the aorta	12	(100.00)
No VSD	11	(91.67)
Normal coronary artery size and origins, bilateral	12	(100.00)
Normal LV systolic function and heart size	12	(100.00)
PDA		
PDA: 0.19 cm	1	(8.33)
PDA closed	4	(33.3)
PDA: 0.11 cm	1	(8.33)
PDA: 0.12 cm	1	(8.33)
PDA: 0.13 cm	1	(8.33)
PDA: 0.17 cm	1	(8.33)
PFO vs. ASD, secundum, left-to-right shunt	12	(100.00)

PFO, patent foramen ovale; ASD, atrial septal defect; PDA, patent ductus arteriosus; TR, tricuspid regurgitation; PR, pulmonary regurgitation; PA, pulmonary artery; LV, left ventricle; RV, right ventricle.

## Data Availability

The original contributions presented in the study are included in the article, further inquiries can be directed to the corresponding author.
